# In vitro and in vivo biological activities of azulene derivatives with potential applications in medicine

**DOI:** 10.1007/s00044-021-02701-0

**Published:** 2021-01-30

**Authors:** Paweł Bakun, Beata Czarczynska-Goslinska, Tomasz Goslinski, Sebastian Lijewski

**Affiliations:** 1 Chair and Department of Chemical Technology of Drugs, Poznan University of Medical Sciences, Grunwaldzka 6, 60-780 Poznan, Poland; 2 Chair and Department of Pharmaceutical Technology, Poznan University of Medical Sciences, Grunwaldzka 6, 60-780 Poznan, Poland

**Keywords:** Azulene, Chamazulene, Conjugates, Medicinal chemistry, Molecular consortia

## Abstract

Azulene is an aromatic hydrocarbon that possesses a unique chemical structure and interesting biological properties. Azulene derivatives, including guaiazulene or chamazulene, occur in nature as components of many plants and mushrooms, such as *Matricaria chamomilla*, *Artemisia absinthium*, *Achillea millefolium*, and *Lactarius indigo*. Due to physicochemical properties, azulene and its derivatives have found many potential applications in technology, especially in optoelectronic devices. In medicine, the ingredients of these plants have been widely used for hundreds of years in antiallergic, antibacterial, and anti-inflammatory therapies. Herein, the applications of azulene, its derivatives and their conjugates with biologically active compounds are presented. The potential use of these compounds concerns various areas of medicine, including anti-inflammatory with peptic ulcers, antineoplastic with leukemia, antidiabetes, antiretroviral with HIV-1, antimicrobial, including antimicrobial photodynamic therapy, and antifungal.

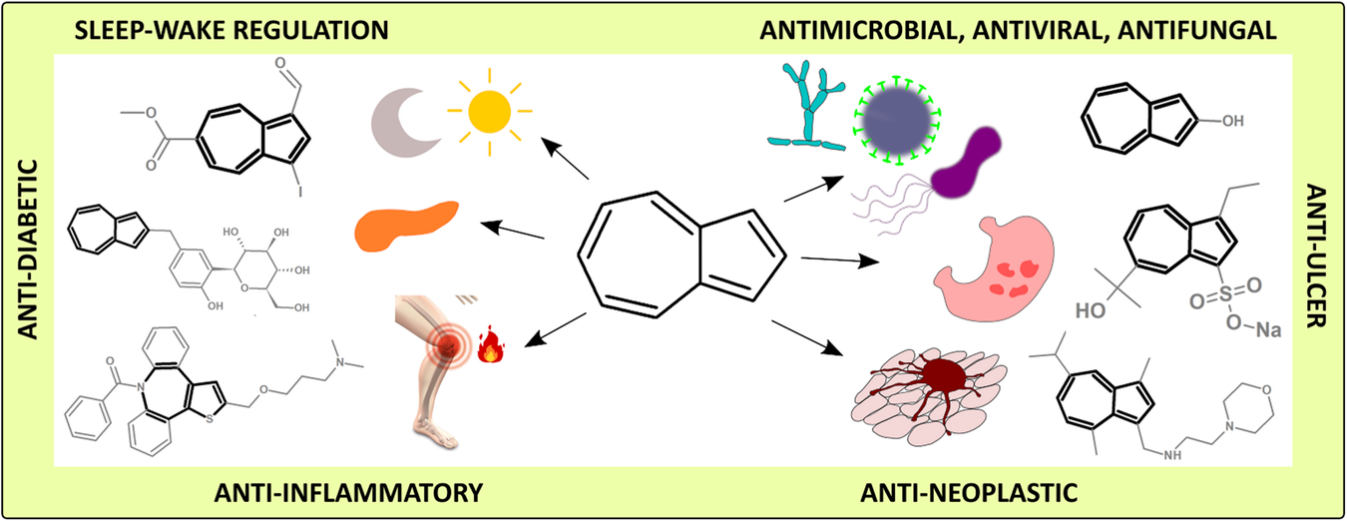

## Introduction

**Fig. 1:**
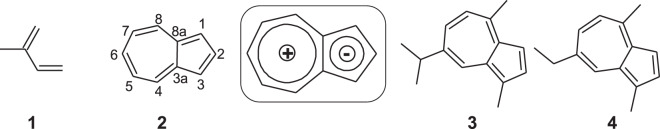
Isoprene moiety **1**, azulene **2**, and its derivatives - guaiazulene **3**, chamazulene **4**. The inset presents unsymmetrical charge distribution in azulene

Terpenes constitute a large family of organic compounds produced mainly in biochemical processes, starting from isoprene moiety **1** (Fig. 1). Examples of great importance in the animal world are carotenoids and vitamin A, which play a crucial role in numerous highly advanced biochemical processes in the human body. Vitamin A is known for improving many functions, including vision, gene transcription, immunity, hematopoiesis, embryonic development, and reproduction [1–4]. Another terpenic compound is squalene, a precursor of steroids, such as cholesterol, bile acid, vitamin D, and steroid hormones. A critical biochemical pathway that allows organisms to produce cholesterol-engaging squalene is the mevalonate pathway [5].

A source of many biologically-active terpenes are plants. Lycopene is a red carotenoid obtained from tomatoes and has proven to decrease risk factors, like LDL-cholesterol and IL-6, in cardiovascular diseases [6]. Another example is botulin, which is a triterpene revealing anticancer properties [7–10], and also demonstrating an ability to inhibit sterol regulatory element-binding proteins (SREBPs). In this second way, it influences the reduction of biosynthesis of cholesterol and fatty acids [11]. In nature, terpenes also serve as a form of protection for plants from microorganisms, parasites, and herbivores.

Many terpenes exhibit biological activity and are used in the pharmaceutical industry. Essential oils obtained from plants e.g., menthol, thymol, and camphor, are active ingredients in nasal decongestants, chest creams, and cough medicines. They also act as antipruritics and anty-itching agents, due to their analgesic properties. Essential oils can also play a role as penetration enhancers in transdermal drug delivery systems [12, 13]. Triterpenoid compounds like oleanolic acid, ursolic acid, and their derivatives show anti-inflammatory, anticancer, antidiabetic, antioxidant, and antibacterial effects [14, 15]. Noteworthy is paclitaxel, which is an anticancer drug isolated from *Taxus brevifolia*, and is also a diterpenoid belonging to the group of terpenes. What is more, it is approved for breast cancer treatment, AIDS-related Kaposi sarcoma, non-small cell lung cancer, and ovarian cancer [16].

Azulene **2** and its derivatives guaiazulene **3** and chamazulene **4** are aromatic hydrocarbons with unique chemical structure, physicochemical and biological properties. The azulene molecule consists of two condensed rings, cyclopentadiene and cycloheptatriene, and possesses 10π electrons, so it obeys Hückel’s rule. Moreover, azulene with its deep blue color is isomeric with colorless naphthalene and intensively absorbs light in the range of 500–700 nm [17, 18].

Azulene derivatives, due to their extraordinary physicochemical properties, have been proposed for many potential technical applications, such as molecular switching [19], sensors [20, 21], components of various optoelectronic devices [22, 23], and solar cells [24, 25]. However, since azulene compounds originate from medicinal plants, they draw the attention of researchers seeking new active pharmaceutical ingredients (API). Herbs containing terpenes have been applied in medicine since ancient times. Azulene derivatives occur naturally in mushrooms e.g., *Lactarius indigo*. Chamazulene can be found in camomile, wormwood, or achillea oil and is known for antiallergic, antibacterial, and anti-inflammatory applications. These compounds have also revealed themselves to have some cosmetic uses, especially in soothing creams and ointments [17]. Chamomile oil oleogel formulation has been proven to be efficient against many different ailments, including pain, nausea, and vomiting. Photophobia and phonophobia occurring during migraine without aura were also effectively treated. Both of the aforementioned effects were confirmed in a randomized, double-blind, placebo-controlled clinical trial [26]. Guaiazulene, as an ingredient of local pomade, has provided rapid recovery in risky neonates with recalcitrant diaper dermatitis without any side effects [27].

**Fig. 2:**
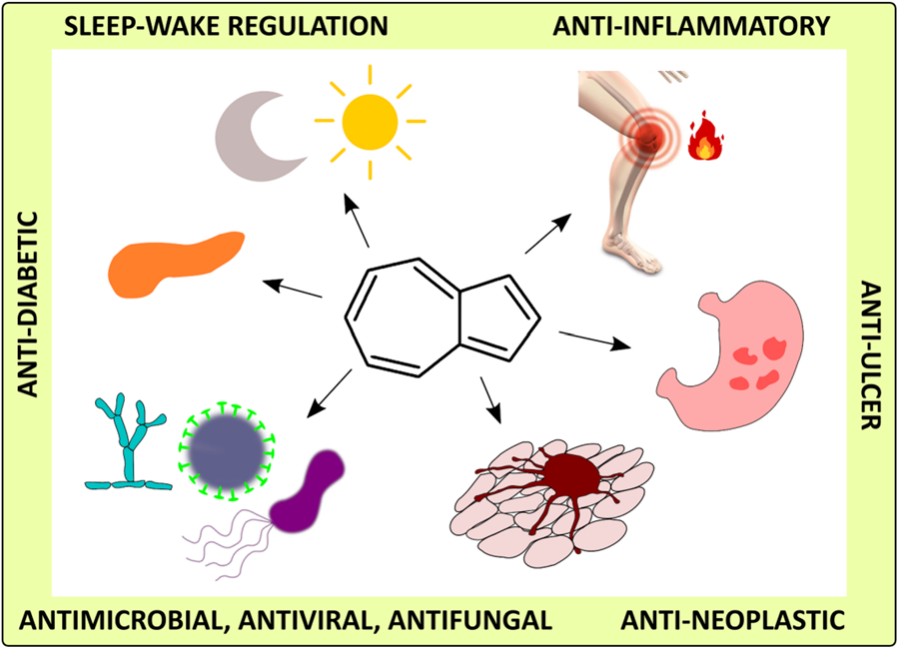
Azulene, its derivatives, and potential applications in medicine

Azulene is a very interesting scaffold for medicinal chemistry applications as it resembles bicyclic aromatics existing in many drugs. Moreover, it can be considered a structural isomer of naphthalene [28]. Modifications of azulene and its derivatives rely on substituents’ addition or their conjugation with other biologically active molecules. Introducing substituents to the core of terpene can increase its solubility, modify absorption, and metabolism. However, the idea of its combining with other active compounds, pharmacophores, or moieties can lead to more profound results. The concept of creating molecular conjugates, hybrids, dimers, and other consortia brought drug discovery science to an entirely new unexplored dimension, which has been lately reviewed by Pawełczyk et al. [29]. Therefore, we present different azulene derivatives and conjugates, including selected molecular consortia, which exhibite attractive potential for medicine and pharmacy. Potential applications of these compounds’ concern various areas of medicine, including anti-inflammatory with peptic ulcers, antineoplastic with leukemia, antidiabetes, antiretroviral with HIV-1, antimicrobial with antimicrobial photodynamic therapy, and antifungal (Fig. 2).

## Antiviral and antimicrobial activity

**Fig. 3:**
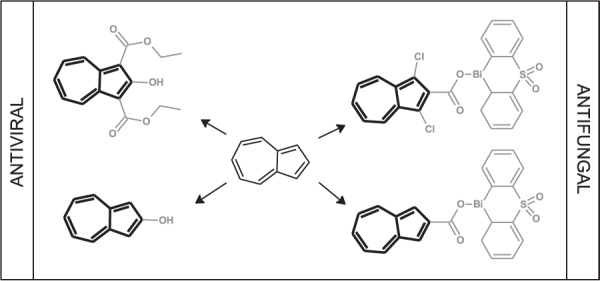
Possible modifications of azulene derivatives of antiviral and antimicrobial potential

One of the greatest achievements of mankind in the field of medicine was the invention of antibiotics about 100 years ago. Recent years have not revealed many new discoveries in the field of new antibacterial drugs, and more bacterial strains resistant to available antibiotics have appeared. Therefore, there is an increasing risk that the advent of the post-antibiotic era will see decreasing access to new drugs and therapies against pathogenic microorganisms [30]. Another significant problem the world has to tackle is how to deal with outbreaks of dangerous viruses in our environment, which affect human life such as AIDS or epidemics like Spanish flu in 1918, SARS in 2002–2004, MERS in 2012, and COVID19 in 2019–2021 [31]. Because of the above-mentioned problems, the development of new antibacterial and antiviral compounds is crucial. It seems that the azulene derivatives presented below could provide new insights and solutions to these issues. Various modifications of the azulene system of potential antiviral and antimicrobial activity are discussed below (Fig. 3).

As an introduction to the application of azulene and its derivatives in antimicrobial photodynamic therapy (PACT), some observations on the toxicity of these molecules, mainly related to the revealed phototoxic properties, should be considered. Such a study was performed by Chiang et al., who examined these natural products in terms of generation of singlet oxygen and other reactive oxygen species (ROS) after excitation with UVA light [32].

Damrongrungruang et al. investigated azulene’s effect on peripheral blood mononuclear cells (PBMCs) viability and singlet oxygen formation in vitro [33]. They found out that azulene at concentrations 5–500 µM when activated with light-emitting diode at 625 nm and light dose 4.2 J/cm^2^ induced singlet oxygen generation. Another conclusion was that PBMCs viability was significantly reduced by azulene at a concentration of 15 µM and red light densities of 4.2, 100, or 200 J/cm^2^. The suggested mechanism of photodynamic reaction with azulene as photosensitizer was proceeding via the generation of ROS, which further caused damage to intercellular structures, including DNA. The authors noted that a few improvements are necessary for further development of PDT in medicine, especially the length of irradiation time. The effects of azulene-light-oxygen action were analyzed in PACT study by Hayek et al. [34]. They performed microbial reduction in ligature-induced periimplantitis in dogs using azulene as a photosensitizer in photodynamic therapy approach and found it equally efficient as conventional infection treatment. Also, PACT was found to be less invasive in comparison to other approaches. This study showed that *Prevotella sp*., *Fusobacterium sp*., and *Streptococcus beta-haemolyticus* were significantly reduced in both groups. During the treatment, 400 µL of azulene 25% solution w/v was applied in the form of a paste based on 10% urea peroxide, 15% tween 80 in 75% carbowax as a vehicle. The 0.01% (w/w) concentration of azulene in the paste was situated on scanned surfaces and exposed to diode GaAlAs laser operating at the wavelength of 660 nm and light dose 40 mW for 180 s. The authors did not note any therapeutic effect without light activation, which indicates that azulene antimicrobial properties rely on photodynamic mechanisms. After treatment, no significant differences were observed between the groups, which suggests that photodynamic therapy is a noninvasive method that could be used to reduce microorganisms in peri-implantitis. Recently, Nagai et al. demonstrated that azulenocyanine revealed the photodynamic antimicrobial effect on human infected dentine plates [35]. In the study, dentin plates extracted from human molars were infected through immersion in a solution of *Streptococcus mutans*. In the treatment procedure with 0.01% azulenocyanine (0.1 ml) after 5 min period in darkness, the 1.5 W laser irradiation at 940 nm revealed the best results in terms of bactericidal effects by reduction of the colony count assay (CFU/ml) and by the ATP assay [35].

**Fig. 4:**
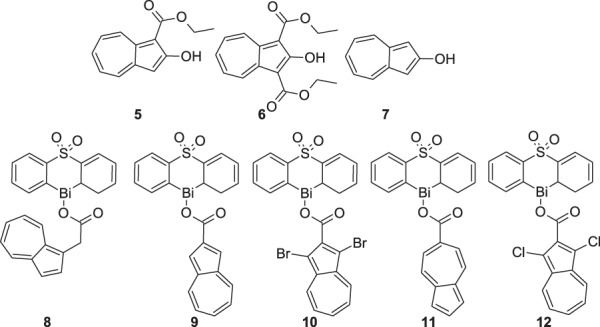
Chemical structures of 2-hydroxyazulene derivatives **5–7** and heterocyclic organobismuth(III) carboxylates with azulene moieties **8–12**

Despite photodynamic studies presented above, azulene and its derivatives demonstrated their own potential against viruses and microbes. In the study performed by Peet et al., 18 azulene derivatives were subjected to in vitro cytotoxicity and anti-HIV-1 assessment [36]. The authors researched the cytotoxicity of the compounds on the U2OS cell line. The least toxic compounds were examined in the first screening phase of the antiviral activity test using HIV-1-based virus-like particles for safe, sensitive, and fast evaluation of the antiretroviral activity. The highest potential was found for 2-hydroxyazulenes **5–7** (Fig. 4), which were selected for the second phase of antiviral test to develop the inhibition of infectious HIV-1 virus replication TZM-bl cells. Finally, compound **5** was not active against the HIV-1 virus, but azulene derivatives **6** and **7** were recognized as active with the best score for **7** with IC_50_ at 8.5 µM and selectivity index 7. The antiretroviral activities of azulene derivatives **5–7** were explained by the inhibition of reverse transcriptase. However, the authors did not exclude other antiviral mechanisms. Following the obtained results, the azulene derivatives can be considered as potential candidates for the development of antiretroviral agents.

The antimicrobial study was based on selected fungi and bacteria. Especially interesting was the study on the antifungal potential of azulene-organobismuth(III) carboxylates. Murafuji et al. synthesized a series of heterocyclic organobismuth(III) carboxylates, including azulene derivatives, and conducted qualitative antifungal assay on the yeast *Saccharomyces cerevisiae* W303-1A with DMSO in a parallel experiment as a negative control [37]. The highly lipophilic compounds, represented by **8–12**, exhibited a satisfactory level of inhibition of fungal growth (Fig. 4). The cationic heterocyclic bismuth-containing scaffold was responsible for inhibitory activity, whereas the carboxylate anion positively affected the drug absorption and bioavailability.

## Anti-inflammatory activity

**Fig. 5:**
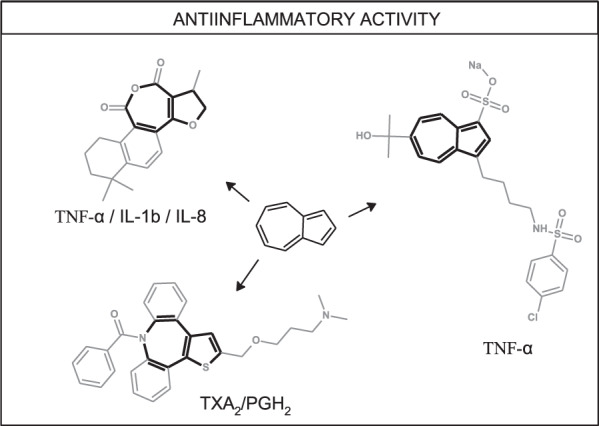
Possible modifications of azulene derivatives of anti-inflammatory potential; TNF-α - tumor necrosis factor α, IL-1b/IL-8—interleukins 1b and 8, TXA_2_/PGH_2_—thromboxane A_2_/prostaglandin H_2_

Inflammation is a physiological reaction of an organism exposed to stress factors such as tissue damage, injuries, cancers, and arthritis. In case of minor contusions and sports injuries, especially in young people, inflammation is even welcome because it helps tissues heal [38]. However, when inflammation becomes chronic, some therapeutic actions are needed. The two most important groups of anti-inflammatory drugs are nonsteroidal anti-inflammatory drugs (NSAIDs), working through cyclooxygenase (COX) inhibiting mechanism, and glucocorticoids, which suppress the immune response and inhibit the synthesis of two main products of inflammation: prostaglandins and leukotrienes. Unfortunately, both groups cause many side effects, and because of that, new approaches in the therapy of chronic inflammation are considered necessary. Various modifications of azulene derivatives of potential anti-inflammatory activity have already been obtained and are discussed below (Fig. 5).

Plants traditionally used in medicine, e.g., camomile (*Matricaria chamomilla*) and yarrow (*Achillea millefolium*), exhibit an anti-inflammatory effect due to the presence of azulene derivatives. Camomile extract was proved to interfere selectively with the COX-2 pathway similarly to the drug sulindac [39]. Moreover, a study performed by Ma and coworkers showed that chamazulene significantly protects against complete Freund’s adjuvant-induced osteoarthritic inflammation in a rat model [40]. Therefore, chamazulene can be recommended as a therapeutic agent for clinical trials against osteoarthritic inflammation. Also, the combinations of azulene derivatives with various molecules reveal attractive anti-inflammatory potential. For example, Nihei et al. studied the efficacy of a combination of sodium azulene sulfonate and L-glutamine to treat oral mucositis caused by anticancer drugs [41]. Patients using sodium azulene sulfonate L-glutamine suspension as a mouthwash three times a day, reported fewer or less severe cases of mucositis in comparison to a control group. Considering its high level of safety and lack of alternatives available to fight oral mucositis induced by anticancer treatment, this procedure seems to offer the chance for the patients to have a better quality of life while fighting cancer.

**Fig. 6:**
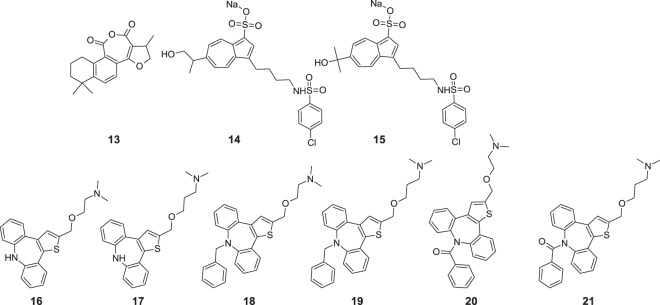
Chemical structures of **13–21**

Azulene scaffold can be encountered among compounds isolated from *Salvia miltiorrhiza var. alba* roots, which are used in traditional Chinese medicine. Ma et al. evaluated the anti-inflammatory activities of tanshinones isolated from *Salvia miltiorrhiza* var. *alba* roots in THP-1 macrophages [42]. These compounds have been applied in the treatment of cardiovascular diseases in local clinical practice. Hexahydrotrimethylnaphthoazulenedione **13** showed anti-inflammatory potency to inhibit the production of TNF-α, IL-1β, and IL-8 in lipopolysaccharide-activated THP-1 macrophages (Fig. 6).

Related potential applications of azulene derivatives were presented by Yokota et al., who focused on finding thromboxane A_2_/prostaglandin H_2_ antagonists with potential use in the treatment of myocardial ischemia, asthma, or peptic ulcer [43]. They obtained a series of new azulene-based compounds and conducted two in vitro experiments on isolated rat aorta and platelet-rich rabbit plasma. These tests enabled SAR analysis and selection of 6-mono- and 6-dihydroxylated-isopropylazulene derivatives of 4-chlorobenzenesulfonamide 3-substituted azulene derivatives for the in vivo experiments in mice. The results of the in vivo experiment showed that pre-treatment with **14** and **15** in a 3.0 mg kg^−1^ p.o. dose prevented U-46619-induced sudden death in mice.

Ozimec Landek et al. synthesized two series of azulene compounds fused with two benzene rings [44, 45] and analyzed the influence of new compounds on tumor necrosis factor α (TNF-α) production. TNF-α can be considered an important inflammatory factor. In these series of derivatives, two carbon atoms of azulene scaffold were replaced with heteroatoms, mainly sulfur. Both series of 1-thia- and 2-thiaazulene derivatives showed in vitro potency to inhibit TNF-α production in lipopolysaccharide activated human peripheral blood mononuclear cells assay. Compounds **16–21** belonging to series with aminoalkoxy chain at C-2 position showed potency to inhibit TNF-α production in vitro in low micromolar range with IC_50_ values for the most potent compounds in the range of 1–3 µM. However, none of these compounds exhibited the ability to inhibit other important inflammation factors like p38 kinase or COX-2 enzyme.

## Antiulcer activity

**Fig. 7:**
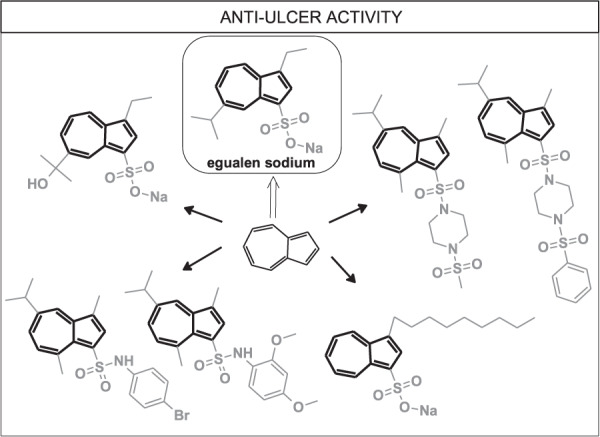
Possible modifications of azulene derivatives of antiulcer potential

Peptic ulcer disease is a prevalent disorder reported by patients and physicians. It is estimated that about 4% of the population suffers from peptic ulcers, and about 10% will develop it during their lifetime [46]. One of the most important groups of drugs used in the treatment of peptic ulcers is called proton-pump-inhibitors (PPIs) and comprises molecules like omeprazole, pantoprazole, and lansoprazole. PPI is the most effective group of drugs used to diminish hydrochloric acid release in the stomach. Even PPIs, which are considered safe, cause side effects. Therefore, new classes of antiulcer drugs are desirable. The success of Egualen, which is a sodium salt of 3-ethyl-7-propan-2-yl-azulene-1-sulfonic acid applied in the treatment of gastric ulcers in Japan under the tradename Azuloxa [47], shows that there is enormous potential for azulene scaffold. Various modifications of the azulene system of potential antiulcer activity have already been performed and are discussed below (Fig. 7).

**Fig. 8:**
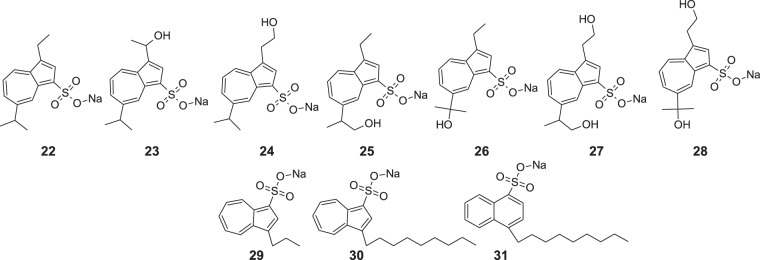
Chemical structures of **22–31**

Yanagisawa et al. studied the anti-peptic activity of compounds related to the metabolites of known API—sodium 3-ethyl-7-isopropyl-1-azulenesulfonate (**22**, KT1-32, egualen sodium) (Fig. 8) [48]. They synthesized six compounds (**23–28**), of which five molecules **24–28** containing 3-alkyl-1-azulenesulfonate moiety are the metabolites of **22** and assessed their antiulcer activity. Compounds **22** and **23** were treated as reference compounds in the study. Metabolites **24–28** showed lower anti-peptic activity than **22**, whereas the lowest activity was revealed by compounds **27** and **28**.

Fujio et al. obtained a very similar compound **30** to the series obtained in the above-discussed study [49]. The difference was the length of the carbon chain in the 3rd position. Afterward, they assessed the surface and antipeptic activity of **30** in comparison to reference molecule **29.** Results of surface properties included the following parameters: critical micelle concentration (CMC), surface tension at CMC (γ_CMC_), efficiency in surface tension reduction (pC_20_, the negative logarithm of the surfactant concentration required to reduce the surface tension of solvent by 20 nN;m^−1^). It was found that **30** exhibits higher antipeptic activity than a reference compound **29**, considering the CMC and IC_50_ (concentration which inhibits peptic activity by 50%) parameters with values such as 0.31 and 0.91 mmol kg^−1^, respectively. The observed activity can be explained as the hydrophobic binding of the amphiphilic molecule **30** to the pepsin’s hydrophobic active pocket. To sum up, the new compound **30** presented slightly less surface activity than its structural isomer 4-decyl-1-naphthalenesulfonate **31**, probably due to the dipole moment within the azulene moiety. Moreover, **30** exhibited higher antipeptic activity than **29**, below half of the CMC, above which level the antipeptic activity of **30** was weakened, probably as a result of the unfolding of bovine serum albumin, which promotes its digestion by pepsin.

**Fig. 9:**
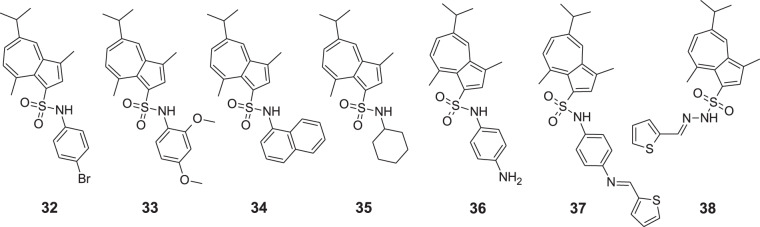
Chemical structures of **32–38**

Zhang et al. synthesized another series of azulene derivatives, which present 5-isopropyl-3,8-dimethylazulene conjugated to arylamines **32–38** (Fig. 9) [50]. The antigastric ulcer activity was studied in vivo on 200 Kunning mice with ethanol-induced gastric ulcers. The structure–activity relationship (SAR) of new compounds was investigated in mice which were treated with an oral dose of suspension containing in its composition carboxymethyl cellulose sodium as an additive and tested compounds—test group, omeprazole—drug reference group, precursor guaiazulene sulfate sodium (GAS-Na), and 5% of carboxymethyl cellulose sodium alone—control group. The oral route’s applied dose of suspension was administered in an amount of 0.4 mL/20 g once a day for five consecutive days. Thirty minutes after applying the last dose, mice were given 0.5 mL of anhydrous ethanol orally, and then after a further 30 min, the animals were euthanized. Subsequent gastrectomy allowed for the assessment of ulcerative lesions. The research demonstrated that compounds **32–38** can be considered as promising antigastric ulcer agents. Moreover, a comparison of arylamino-substituted compounds with alkylamino substituted compounds showed advantages of the first group over the second for antiulcer effectiveness. The results of the study gave reason for optimism as some molecules presented the best antiulcer activity with ulcer index values lower than that observed for the reference drug omeprazole.

**Fig. 10:**
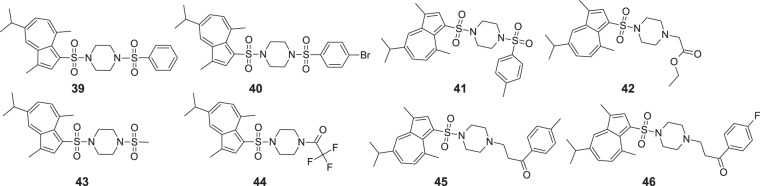
Chemical structures of **39–46**

Cao et al. studied antigastric ulcer activity in vivo of guaiazulene derivatives on 221 mice divided into 28 groups [51]. They obtained guaiazulene sulfonate derivatives with a double sulphonylamino motif due to piperazine presence and studied their antigastric ulcer activity through an ethanol-induced gastric ulcer model method. All studied guaiazulene derivatives presented antigastric ulcer activity. It is worth noting that compounds **39–46**, showed a significant reduction of gastric ulcers, whereas the most promising compounds **39**, **42**, **43** exhibited even better antiulcer activity than the first-line drug omeprazole (Fig. 10).

The researchers also provided SAR analysis. Many parameters such as topological polar surface area (TPSA), octanol–water partitioning coefficient (log *P*), molecular molar refractivity (MR), calculated partition coefficient (Clog P), molecular weight (MW) were taken into account and compared. Unfortunately, there were not enough data to prove the correlation between the mentioned parameters and biological activity. Nonetheless, the authors noted some dependencies. For example, compounds possessing two sulphonylamino moieties revealed better activity than other compounds. In general, derivatives with electron-withdrawing substituents exhibited better activity than molecules with electron-donating groups, whereas the bromine element played an important role in enhancing activity. The mechanism of action of guaiazulene derivatives is not known at the moment. Scientists proposed two probable explanations: surface coverage and multifunctional group synergistic mechanisms.

## Antineoplastic activity

Cancers and other neoplasms are still a significant health threat with high expenditure for cancer care, which in the USA in 2018 reached ca. 150 billion dollars. Moreover, cancer is still one of the leading causes of death worldwide, despite extensive research on new antineoplastic medications. In 2018, there were more than 18 million new cases and more than 9 million cancer-related deaths worldwide. Approximately 39.5% of men and women will be diagnosed with cancer at some point in their lives [52]. Regardless of the progress of science and medicine, there is a great need for new treatment options for these diseases. To fight this ubiquitous threat, scientists work on sophisticated strategies such as retrospection based on traditional medicine and herbal remedies, variations with light in photodynamic therapy, and nanomedicine.

**Fig. 11:**
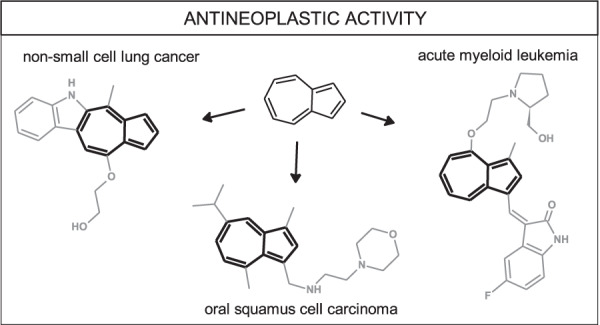
Possible modifications of azulene derivatives of anticancer potential

Azulene moiety, as an element of molecular consortia, raises great hope for effective anticancer drugs. Photodynamic activation with red laser light and guaiazulene revealed the generation of singlet oxygen and suppression of inflammatory markers in peripheral blood mononuclear cells [53]. The chamazulene/camphor-rich essential oils containing 63% of chamazulene from *Artemisia arborescens* plants collected in Sicily showed promising activity on studied melanoma cancer cells with potential to trigger apoptotic death [54]. It is worth noting that, according to some studies, chamazulene is an even more potent antioxidant than ascorbic acid and α-tocopherol [55]. Various modifications of azulene system of potential antineoplastic activity, which have already been performed, are discussed below (Fig. 11).

**Fig. 12:**
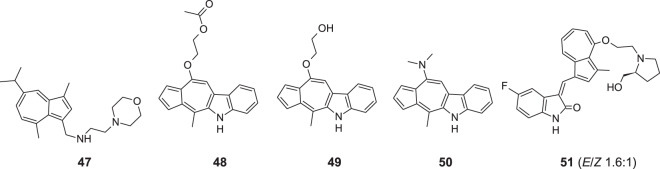
Chemical structures of **47–51**

Uehara et al. obtained a series of 10 alkylaminoguaiazulene derivatives towards both cancer and normal cells [56]. Cytotoxicity was assessed using the 3-(4,5-dimethylthiazol-2-yl)-2,5-diphenyltetrazolium bromide (MTT) method. Moreover, apoptosis-inducing activity was evaluated by cleavage of poly ADP-ribose polymerase and caspase-3 with western blot analysis. It was found that with the increasing length of the alkyl group of alkylaminoguaiazulene derivatives, cytotoxicity increased, whereas the introduction of oxygen, nitrogen, or sulfur atom into the alkyl group slightly reduced cytotoxicity. Among compounds studied, guaiazulene substituted with morpholine **47** showed very high tumor specificity, as well as apoptosis-inducing activity against oral squamous cell carcinoma (Fig. 12). In another study, alkoxyl guaiazulene-3-carboxylates were also considered as potential candidates for anticancer medicines or leading structures for further derivatization [57].

Hong et al. synthesized a group of 14 azulene-indoles using a microwave-assisted method [58]. After that, an evaluation of their in vitro activity against 60 human cancer cell lines, including melanoma, leukemia, lung, colon, renal, ovarian, central nervous system, breast, and prostate lines, was performed according to standard National Cancer Institute protocols. Results indicated that compound **49** could be considered a potent inhibitor of EKVX non-small cell lung cancer cell line growth with IC_50_ 1.00 µM and HS578T breast cancer cell line with IC_50_ 0.93 µM. Also, other compounds **48** and **50** showed interesting antineoplastic activity. Molecule **48** was found a selective and potent growth inhibitor of K-562 leukemia cancer cell line with IC_50_ 0.51 µM, and revealed good inhibition properties in HOP-92 non-small cell lung cancer cell line with IC_50_ 1.38 µM. Azulene derivative **50** presented good potential against HOP 92 cell line with IC_50_ 0.56 µM and PC-3 prostate cancer cell line with IC_50_ 1.41 µM. The mechanism of action of the compounds mentioned above was not indicated.

A very interesting study on a series of azulene derivatives as potent multi-receptor tyrosine kinase inhibitors for the treatment of acute myeloid leukemia (AML) or gastrointestinal stomach tumors (GIST) was performed by Chen et al. [59]. After synthesis and screening tests, compound **51** presented high cytotoxicity against human peripheral blood cell line MV4-11 with IC_50_ values in the submicromolar range. Simultaneously, it also inhibited KDR, cKIT, PDGFRβ, and FLT-3 receptors. Afterward, scientists analyzed the pharmacokinetics of **51** in rats and measured the essential parameters after administering its single intravenous or oral dose. The next experiment was conducted on human leukemia MV4-11 xenografts in BALB/c nude mice and revealed that on the 30th day after the beginning of treatment, using 50 mg/kg dose twice a day, tumor growth inhibition by **51** was 99 ± 0.82%. The results indicate that compound **51** has a great potential to become a perspective antileukemia agent, and it can undoubtedly move to the next phase of research—clinical trial. Therefore it can be considered as a potent FMS-like tyrosine kinase 3 inhibitor and antileukemia agent.

## Antidiabetic activity

**Fig. 13:**
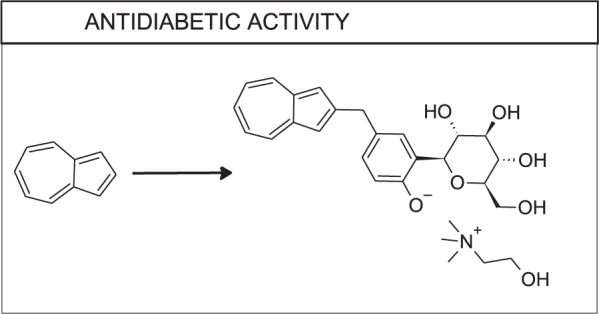
Possible modifications of azulene derivatives of antidiabetic potential

Diabetes is a prevalent metabolic disorder characterized by insulin resistance and persistent hyperglycemia. Each year nearly 85 thousand USA residents die due to diabetes, according to the Centers of Disease Control and Prevention U.S. Department of Health & Human Services [60], thus making diabetes the 7th most common cause of death in the USA. Many research groups worldwide do their best to propose new, more efficient, and patient-friendly therapies for this disease. Various modifications of the azulene system of potential antineoplastic activity, which have already been performed, are discussed below (Fig. 13).

**Fig. 14:**
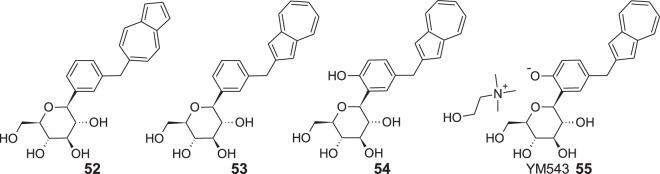
Chemical structures of **52–55**

For this purpose, Ikegai et al. synthesized a series of C-glucosides with azulene in the aglycon moiety, starting from a previously examined compound **52** [61]. Expected inhibitory activity toward human sodium-glucose transport protein 1 (hSGLT1) and human sodium-glucose transport protein 2 (hSGLT2) was evaluated in vitro using Chinese hamster ovary cells (CHO) expressing hSGLT1 or hSGLT2. The researchers measured IC_50_ values regarding to hSGLT2 and selectivity versus hSGLT1 of the essential compounds: **52** (99 nM, 140 fold), **53** (22 nM, 590 fold), **54** (8.9 nm, 280 fold) in at least two independent experiments (Fig. 14). Moreover, preclinical tests on KK/A^y^ type 2 diabetic mice were conducted to confirm the antihyperglycemic effect of **53** in vivo. This test showed that a single oral administration of **53** with a dose 3 mg/kg reduced blood glucose levels significantly by 46% compared to the vehicle. Afterward, the optimization of molecule **53** was performed, leading to the synthesis of **54**, which was finally chosen as the most promising compound from the whole series and proposed to reach clinical trial in the form of mono choline salt **55**, named YM543. Pharmacokinetics studies on normal rats were carried out to assess this compound in the living organism. Results obtained after administering a single 1.0 mg/kg dose intravenously or a single 3 mg/kg dose orally revealed fast oral absorption with *T*_max_ 0.5 h, bioavailability 29%, clearance 2483 L/h/kg, the volume of distribution 3360 L/kg. Renal drug concentration was 23 times higher than that in plasma. Oral administration of 0.3–3.0 mg/kg dose of **54** resulted in a dose-dependent increase in urinary glucose excretion that lasted over 12 h for the dose of 0.3 mg/kg or higher. Moreover, the compound exhibited potent and long-lasting antihyperglycemic activity in both streptozotocin-induced diabetic rats (STZ rats, type 1 diabetes model) and KK/A^y^ mice without decreases into the hypoglycaemic range.

## Various cardiac, vascular, and neurological disorders

**Fig. 15:**
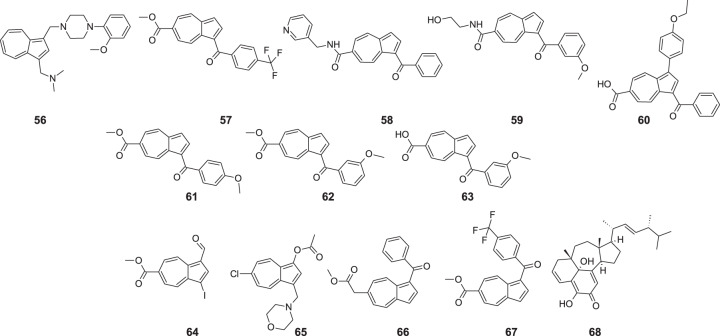
Chemical structures of **56–68**

An interesting study was provided by Löber et al., who developed the synthesis path of azulene derivatives and assessed their affinities for the monoaminergic G protein-coupled receptors, including dopamine, serotonin, histamine, α-adrenergic receptor subtypes, and performed SAR analysis [62]. The authors synthesized a series of azulenylmethylpiperazines inspired by previously examined compound FAUC 3019, a partial agonist of dopamine D_4_ receptor with a potential application in therapy of erectile dysfunction. Receptor binding properties were tested in vitro on Chinese hamster ovary cells. In vivo test of the most promising molecule **56** (Fig. 15) was conducted on 32 male rats. Compound **56** proved to induce penile erection in male rats after systemic (subcutaneous, sc) administration and injection into the paraventricular nucleus (PVN). In low concentrations, the effectiveness of **56** was better than that of the reference—apomorphine. Azulene derivative **56** revealed excellent D_4_ receptor affinity, good selectivity, and was considered as D_4_ partial agonist with EC_50_ at 0.41 nM. The rationale for incorporation of azulene moiety into novel compounds can be explained by the specific charge distribution within this molecule with a significant negative molecular electrostatic field (MEP) below and above the five-membered ring, and a positive MEP at the seven-membered ring (Fig. 1). Moreover, the large dipole moment of azulenes made molecule **56** easily recognizable for the dopaminergic D_4_ receptor. The results proved that the new compound **56** could be considered for further study towards erectile dysfunction. Nevertheless, more study in this field is needed, including comparative research on the effectiveness and safety profile of newly synthesized compound **56** and already approved for the treatment of erectile dysfunction with first-line drugs like sildenafil and tadalafil.

Turku et al. used in silico analytic software to propose compounds for synthesis of orexin (hypocretin) OX1 and OX2 receptor ligands [63]. As the receptor is involved in the sleep–wake regulation, the antagonists are considered to treat insomnia. Moreover, the agonists are suspected of becoming useful in treating daytime sleepiness, narcolepsy, and certain cancer types. Besides, some ligands can act as potentiators with expected application in orexin receptor activation therapies when the production of endogenous peptides is not fully terminated. In the research, seven potentiators and two weak dual orexin receptor agonists were found. The activity assessment consisted of monitoring Ca^2+^ response in Chinese hamster ovary (CHO)-K1 cells heterologously expressing high densities of human OX_1_ and OX_2_ receptors. Calcium ions elevation is considered to be a prominent response to orexin receptor activation. The study confirmed that compounds **57–60** acted as weak orexin receptor agonists, whereas compounds **61–63** potentiated the response to orexin-A on OX_1_ receptors by 1,4-, 1,6- and 1,3-fold, respectively, at 10 µM. The most critical finding of SAR analysis was the observation that agonism and potentiation are related to the presence of bicyclic aromatic ring moiety substituted with two hydrogen bond acceptors (1-position, benzoyl; 6-position, carboxyl/ester) within 7–8 Å of each other. Moreover, it was found that subtle changes in the structure cause changes in the activity, possibly because the overlapping of binding sites occurs. It seems an interesting research direction, but to even think about the future medical application of mentioned compounds, more experiments are necessary, especially toxicology assessment and pharmacokinetics in vivo.

The orexin system is critical in terms of sleep–wake regulation, stress response, and insomnia. Therefore there is interest in small molecular potentiators that enhance the effects of endogenous neuropeptides, especially orexin-A and orexin-B. In another study, Leino et al. screened a library of 70,000 synthetically accessible azulene-based compounds targeting orexin-based receptors [28]. The most promising molecules **64** and **65** showed *K*_i_ values in the low micromolar range 3–9 µM, whereas compounds **66** and **67** acted as weak OX receptor agonists. Three other compounds exhibited a concentration-dependent potentiation of response to orexin-A at the OX_1_ but not OX_2_ receptors with twofold potentiation at 10 µM.

Although new azulene derivatives come from synthetic reactions in the laboratory, the plethora of their derivatives are still present in various plants. An excellent illustration of this is ergosterol ganotheaecolin A (**68**), found in mushroom *Ganoderma theaecolum* by Luo et al. [64]. The fungus is commonly used in traditional Chinese medicine for the treatment of neurological disorders. After isolation and confirmation of structure, scientists conducted for this molecule in vitro evaluation of neurite outgrowth-promoting activity in PC12 cells, using NGF as control. The results indicated that **68** could stimulate cell differentiation in a dose-dependent manner and reached the maximum effect at 10 µM. Much research still needs to be performed in this area, but **68** revealed the potential to become a cure for neurological diseases in the future.

## Conclusion

Terpenes constitute a large family of organic compounds produced mainly in biochemical processes, starting from isoprene moiety. The most important example of this group is vitamin A which is involved in many biochemical processes such as vision, gene transcription, immune function, hematopoiesis, embryonic development, and reproduction. Another important terpenic compound is squalene, a precursor of cholesterol, bile acid, vitamin D, and steroid hormones. In nature, terpenes often serve as plant protection from microorganisms, parasites, or herbivores, and because of that, many biologically-active terpenes are obtained from plants. To the terpene family created by nature belong, among others: lycopene, botulin, menthol, thymol, camphor, paclitaxel, oleanolic acid, ursolic acid, chamazulene, and guaiazulene. Most terpenes reveal a long list of traditional uses in herbal extracts due to their anti-inflammatory, anticancer, antidiabetic, antioxidant, or antibacterial activity.

Azulene, chamazulene, and guaiazulene have been known for ages and widely applied in medicine. In this review, we presented new potential applications of their derivatives in medicine and pharmacy. Azulene scaffold exhibits an interesting perspective for medicinal chemistry as it can be considered a structural isomer of naphthalene. Therefore, introducing the bicyclic azulene hydrocarbon into various molecular consortia leads to compounds with a vast spectrum of biological activities. The azulene derivatives may be potentially applied in the therapy of peptic ulcers, leukemia, lung cancer, diabetes, and various viral, fungal, and microbial infections. In this review, we presented many biological studies, which were performed in vitro on various cell lines, e.g., PBMCs, U2OS, TZM-bl, EKVX, HS578T, K-562, HOP-92, PC-3, MV4-11, CHO, (CHO)-K1, PC12, and in vivo on rats and mice. The most promising applications of azulene derivatives were proposed for the treatment of peptic ulcer disease. It is worth noting that in the biological study, some of the azulene derivatives revealed even better antiulcer activity than a reference drug omeprazole. Another promising biological activity of azulene derivatives was their influence on the growth of selected cancer cell lines e.g., K-562 leukemia cancer cell line with IC_50_ value on the micromolar level. Azulene scaffold also creates perspective applications for diabetes treatment. To sum up, many structures that contain azulene moiety can be considered as candidates for drugs. Nevertheless, more research in the area of toxicity needs to be carried out before moving to clinical trials and the market.

## Abbreviations

AML: acute myeloid leukemia
API: active pharmaceutical ingredient
CHO: Chinese hamster ovary cells
Clog P: calculated partition coefficient
CMC: critical micelle concentration
γ_CMC_: surface tension at CMC
COX: cyclooxygenase
GAS-Na: guaiazulene sulfate sodium
GIST: gastrointestinal stomach tumors
hSGLT1: human sodium-glucose transport protein 1
hSGLT2: human sodium-glucose transport protein 2
IC_50_: the half maximal inhibitory concentration
IL: interleukin
log P: octanol-water partitioning coefficient
MEP: molecular electrostatic field
MR: molecular molar refractivity
MTT: 3-(4,5-dimethylthiazol-2-yl)-2,5-diphenyltetrazolium bromide
MW: molecular weight
NSAIDs: nonsteroidal anti-inflammatory drugs
PACT: antimicrobial photodynamic therapy
PBMCs: peripheral blood mononuclear cells
PGH_2_: prostaglandin H_2_
PPIs: proton pump inhibitors
PVN: paraventricular nucleus
ROS: reactive oxygen species
SAR: structure-activity relationship
SREBPs: sterol regulatory element-binding proteins
STZ rats: streptozotocin-induced diabetic rats
TNF-α: tumor necrosis factor α
TPSA: topological polar surface area
TXA_2_: thromboxane A_2_
